# 874. Impact of Telehealth in HIV Ambulatory Clinic during COVID-19 Pandemic Impact of Telehealth in HIV Ambulatory Clinic during COVID-19 Pandemic

**DOI:** 10.1093/ofid/ofab466.1069

**Published:** 2021-12-04

**Authors:** Sandhya Nagarakanti, Eliahu Bishburg, Donna George, Kristen Ehlers

**Affiliations:** Newark Beth Israel Medical Center, Newark, NJ

## Abstract

**Background:**

HIV outpatient in-person (IN-P) visits were limited during the COVID-19 pandemic, and most patients (pts) were cared for remotely through telehealth (TELE). We sought to evaluate the impact of TELE on HIV infected pts during the pandemic compared to the pre-pandemic IN-P care.

**Methods:**

Retrospective chart review of pts in an outpatient HIV clinic, study period 03/30/2019 to 03/29/2021. Two periods were defined: pre-COVID (Pre-CO) 3/30/2019 to 3/29/2020 and COVID (CO) 3/30/2020 to 3/29/2021. Data was collected on demographics, HIV risk, type of encounter, number of encounters, CD4, HIV Viral loads (VL) at first, and last visit, treatment regimen information. HIV VL < 200 copies/ml was considered as undetectable.

**Results:**

A total of 607 pts were evaluated. Mean age 51years; (Range-20-84). Male 306 (50.4%), African American 545(90%), Hispanic 50 (8.2%), white 9 (1.5%), Asian 3(0.5%). HIV risk: heterosexual 437(72%), male sex with male 118(19.4%), intravenous drug use 8 (1.3%). In the Pre-CO period, 530 pts were seen as IN-P; in the CO period 606 pts were encountered of which 304 (50.2%) were TELE visits, 89(14.7%) IN-P, 213(35%) had both TELE and IN-P encounters. Mean number of encounters were 2.59 in the Pre-CO and 2.46 during CO. The number of new pts in the Pre-CO were 36 (7%) vs. 52(8.6%) in the CO (p=0.26). During the pre-CO, 373 pts had CD4 measured at first and last visits, 353(95%) at the first visit and 352 (94.3%) at the last visit had CD4 counts ≥ 200/uL (p=.87); 373 pts had a VL done at first and last visits, 330 (88.5%) at the first visit and 337(90.3%) at last visit were undetectable (p=0.41). During CO, 445 pts had CD4 measured at first and last visits, 402 (90.3%) at the first visit and 445(94.2%) at the last visit had CD4 count ≥200/uL (p=0.03); 448 pts had VL measured at first and last encounters, 389(87%) at the first visit and 417(93%) in the last visit were undetectable (p=0.002). Antiretroviral changes occurred in 29% in the Pre-Co compared to 19% in the CO (p=0 .32).

**Conclusion:**

In our clinic, more pts were cared for during the CO period compared to the Pre-CO period. Significantly, more pts had undetectable HIV VL during CO period. At least one TELE visit was utilized by over ¾ of the pts. TELE has a potentially important role in future HIV care without compromising patient outcomes.

**Disclosures:**

**All Authors**: No reported disclosures

